# Role of Metabolic Parameters of 18F-Fluorodeoxyglucose Positron Emission Tomography-Computed Tomography (18F-FDG PET-CT) Imaging in Predicting Progression-Free Survival of Radioiodine-Refractory Differentiated Thyroid Cancer: A Single-Center Study

**DOI:** 10.7759/cureus.84998

**Published:** 2025-05-28

**Authors:** Alireza Khatami, Duncan Sutherland, Jonathan Romsa

**Affiliations:** 1 Division of Nuclear Medicine and Molecular Imaging, Department of Medicine, The Ottawa Hospital, University of Ottawa, Ottawa, CAN; 2 Medical Imaging, London Health Sciences Centre, Western University, London, CAN

**Keywords:** metabolic activity, positron emission tomography, progression-free survival, radioactive iodine, thyroid cancer

## Abstract

Introduction: Most cases of thyroid cancer are differentiated thyroid cancers, which typically have a high survival rate due to the effectiveness of radioactive iodine (RAI) therapy. However, a subset of these cancers, known as radioactive iodine-refractory differentiated thyroid cancer (RR-DTC), is resistant to RAI and is associated with lower survival rates, necessitating alternative therapeutic approaches. As RR-DTC develops, there is an increase in glucose utilization and metabolic activity of the tumor. The technique of 18F-fluorodeoxyglucose positron emission tomography-computed tomography (18F-FDG PET/CT) is well-known for assessing the metabolic activity of tumors, and in this case, the RR-DTC. This study explores the relationship between 18F-FDG PET/CT imaging and associated metabolic parameters of RR-DTC to progression-free survival (PFS).

Methods: A retrospective analysis was performed on 22 patients diagnosed with RR-DTC who underwent 18F-FDG PET-CT imaging between 2010 and 2021. Metabolic PET parameters, including total lesion volume (TLV), total lesion glycolysis (TLG), maximum standardized uptake value (SUVmax), and the biomarker thyroglobulin (Tg), along with thyroglobulin doubling time (TgDT), were extracted and analyzed for potential associations with PFS. Means and standard deviations (SD) were reported for continuous variables, and percentages for categorical variables. Student’s t-test and Fisher’s exact test were used to compare imaging parameters and biomarker variables between patients with and without disease progression. Progression-free survival (PFS) was evaluated using univariate and multivariate Cox proportional hazards models, and the Kaplan-Meier method with log-rank test was used to assess the impact of various variables on PFS. All statistical analyses were performed using SPSS software version 28.1.1, with a two-sided significance level set at P < 0.05.

Results: The patients’ ages ranged from 38 to 83 years, 15 out of 22 (68%) were male, and 13 out of 22 (59.1%) exhibited distant metastases. The follow-up period varied from 21 to 452 months; the median follow-up was 32 months, and the mean follow-up was 116 months. Of the 22 patients, 11 (50%) demonstrated disease progression, with a mean time-to-progression of 74 months. The mean SUVmax and TLV were higher in patients with metastatic disease compared to those with localized disease in surgical beds and regional lymph nodes (p-values of 0.045 and 0.01, respectively). Univariate Cox analysis revealed that SUVmax > 10 had a hazard ratio (HR) of 4.97 (CI: 1.39-17.8, p-value = 0.014), TLV > 5 had an HR of 11.6 (CI: 2.51-53.4, p-value = 0.002), Tg > 10 had an HR of 5.70 (CI: 1.44-22.6, p-value = 0.013), and TgDT ≤ 100 days had an HR of 17.9 (CI: 1.89-161.8, p-value = 0.01), all correlated with worse PFS. Multivariate Cox analysis demonstrated that TgDT ≤ 100 days with an HR 63.9 (CI: 9.33- 743, p-value=0.02) was the sole predictor of reduced PFS. Kaplan-Meier analysis showed that SUVmax >10, TLG >10, Tg > 10, and TgDT ≤ 100 days corresponded to worse PFS, and TgDT ≥300 days corresponded to best PFS.

Conclusion: In this data set, the metabolic parameters obtained from PET-CT imaging are predictive for PFS in RR-DTC patients when used with other imaging and biomarkers.

## Introduction

Differentiated thyroid cancer (DTC), which includes papillary and follicular subtypes, is the most common endocrine malignancy, with a steadily increasing incidence. Radioactive iodine (RAI) is shown to positively impact the survival metrics in DTC patients in many previous studies in various geographical areas [1,2]. Following surgery, RAI remains the treatment of choice for moderate- to high-risk DTC. However, a small fraction of DTC cases develop resistance to radioactive iodine (RAI), known as radioactive iodine-refractory differentiated thyroid cancer (RR-DTC). RR-DTC is defined by three criteria: 1) the presence of at least one focus of disease that demonstrates no RAI uptake, 2) disease progression during radioiodine treatment, and 3) persistent disease after receiving a cumulative dose of 600 mCi (22.2 GBq) of radioiodine treatment [3]. RR-DTC carries a worse prognosis, with a 10-year survival rate of less than 10% [4,5], in contrast to the 96% survival rate seen in radioiodine-sensitive differentiated thyroid cancer [6].

Diagnosing and managing RR-DTC presents considerable challenges, often leading to a poor prognosis [7]. Available treatments for RR-DTC include systemic therapy with targeted approaches using tyrosine kinase inhibitors (TKIs), such as Lenvatinib and Sorafenib. These therapies have shown improved PFS among RR-DTC patients, especially when initiated in the early stages of the disease [8,9]. Redifferentiation therapy using high doses of RAI and new agents targeting various genes and enzymatic pathways can also be used for the treatment of this category of thyroid cancer.

Considering the higher recurrence rate and lower PFS in RR-DTC, it is critical that patients receive regular assessment using biochemical and imaging modalities. In the case of CT imaging, the recommended approach, as endorsed by international societies, is the Response Evaluation Criteria in Solid Tumors (RECIST) to assess therapeutic response and follow-up in both DTC and RR-DTC during treatment [10,11].

Conversely, various studies have indicated that biomarkers such as thyroglobulin (Tg) and thyroglobulin doubling time (TgDT) could offer valuable insight during RR-DTC follow-up, including outcome prediction. Tg in particular has been extensively studied in the context of differentiated thyroid cancer [12,13]. Recently, TgDT has attracted significant interest as an additional biomarker and has been thoroughly investigated for DTC [14,15]. However, the efficacy of these markers in forecasting outcomes for RR-DTC remains unclear, in contrast to their established roles in DTC. Furthermore, both Tg and TgDT lose their predictive value in cases where the cancer undergoes differentiation [7].

In RR-DTC, the relationship between Tg or TgDT and CT scan findings is not always consistent with disease progression. Some studies have shown fluctuations in Tg levels that do not necessarily reflect the disease status [16].

Given the challenges of RR-DTC, 18F-FDG PET-CT imaging is endorsed by the American Thyroid Association for the evaluation and management of this condition. This imaging technique is pivotal in demonstrating disease aggressiveness by localizing and assessing disease extent and quantifying metabolic activity within capabilities that may surpass those of other imaging modalities [10,17]. Patients with a positive 18F-FDG PET-CT scan exhibit a sevenfold increase in mortality risk compared to thyroid cancer patients who have a negative scan [18]. Furthermore, research has demonstrated that PET-CT imaging significantly enhances the ability to distinguish between benign and malignant thyroid lesions [19]. Advanced PET techniques extend the risk stratification of RR-DTC beyond mere visual assessment and basic uptake measurements. Investigative efforts have focused on variables such as the standardized uptake value maximum (SUVmax), total lesion volume (TLV) measured in milliliters (mL), and total lesion glycolysis (TLG), calculated as the product of SUV mean and cm³ or TLV [20], which have been applied to thyroid cancer research [21].

The American Thyroid Association and European Thyroid Association guidelines currently recommend the use of 18F-FDG PET-CT imaging for high-risk DTC patients with elevated serum Tg levels (typically >10 ng/mL) but negative RAI imaging results. It may also be considered in the following scenarios: i) as part of the initial staging process for cancers with invasive features, such as Hürthle cell carcinomas, in conjunction with other evidence of disease on imaging or elevated serum Tg levels, ii) as a prognostic tool for identifying lesions and patients at the highest risk for rapid disease progression and disease-specific mortality in the presence of metastatic disease, and iii) for evaluating posttreatment response following systemic or local therapy for metastatic or locally invasive disease [10,22].

Considering the less clearly defined roles of the biomarkers Tg and TgDT in predicting RR-DTC outcomes compared to DTC [7], the current study aims to investigate the metabolic parameters of PET-CT imaging and their correlation with patient outcomes. The objective of this study is to evaluate the prognostic value of 18F-FDG PET/CT metabolic parameters and their correlation with thyroglobulin levels and TgDT in predicting disease progression in patients with RR-DTC.

## Materials and methods

We conducted a single-center, retrospective study of patients diagnosed with differentiated papillary and follicular thyroid cancers (DTC) who underwent thyroid resection followed by post-surgical RAI ablation, and who exhibited signs of radioiodine resistance between December 2010 to June 2021.

The inclusion criteria for this population included: Biochemical failure with rising Tg, despite RAI uptake; development of new lesions on the whole-body iodine scan during RAI therapy; negative whole-body iodine scan and elevated Tg; rising Tg levels following a cumulative dose of 22.2 GBq (600 mCi)of RAI; RAI uptake in some lesions and no uptake in other lesions. In cases where Tg levels were low or undetectable due to the presence of antithyroglobulin antibodies (anti-Tg), an increase in anti-Tg antibody levels was also considered indicative of disease progression.

Exclusion criteria included patients with anaplastic, medullary, undifferentiated thyroid cancer, other non-thyroid malignancies, and pregnant women; patients with DTC who had rising Tg levels but negative 18F-FDG PET-CT scans; patients with locoregional disease who were treated with surgery and RAI or external beam ablation had a complete response; patients who were lost to follow-up.

The study received approval from the local Research Ethics Board (REB).

Biomarker follow-up

Tg level was regularly measured during patient follow-up, with evaluation occurring at least once a year. If there was clinical suspicion of disease progression, the assessment interval was shortened. Tg levels at the time of diagnosis of RR-DTC were used for TgDT evaluation. TgDT calculations were performed using the doubling time and progression calculator developed by Miyauchi et al. For TgDT calculations, at least three, and preferably four, previous Tg values were retrieved from the electronic medical record (EMR) during serum thyroid-stimulating hormone (TSH) suppression and included in the calculator, and the output of the calculator as a number of days for doubling time was included in our spreadsheet. The time between the Tg and 18F-FDG PET-CT imaging was ± one month.

Imaging follow-up

All included patients underwent post-ablation follow-up beginning with a whole-body 131-I scan (post-ablation) and regular neck ultrasounds. If biomarkers remained detectable or increased, CT scans of the neck, chest, and thorax, as well as 18F-FDG PET-CT scans, were performed. The first positive 18F-FDG PET-CT scan was utilized to extract metabolic variables, including SUVmax, TLV, and TLG. The measurement of metabolic parameters on 18F-FDG PET-CT was performed using a gradient segmentation strategy through MIM PET Edge software (Cleveland, Ohio). These measurements were accomplished by employing a gradient segmentation strategy. The gradient segmentation method entails the computation of spatial derivatives along the tumor’s radii. Through this methodology, the boundaries of the tumor were established by evaluating the derivative levels and ensuring the continuity of the tumor’s outline. An operator places an initial marker close to the lesion’s central point. By extending the cursor outward from this center by the operator, six axes are projected in various directions, providing a visual guide for commencing the gradient segmentation. Spatial gradients were dynamically calculated along each axis, and the length of an axis was curtailed when notable spatial gradients were detected. The amalgamation of these six axes formed an ellipsoid, serving as the initial region for detecting gradients in metabolic activity to outline the metabolic tumor volume (MTV) of the lesion. This method was used for all detectable lesions, and a cumulative MTV was calculated by summing up the MTV values of all detectable lesions. This process was applied to all identifiable lesions, and their MTVs were aggregated to determine TLV. Finally, TLG was determined by multiplying the metabolic tumor volume by the mean standardized uptake value (SUV mean) [20].

Clinical follow-up

All patients were monitored for PFS, defined as the time between DTC diagnosis and the confirmation of RR-DTC by 1) evidence of biochemical progression on blood work, 2) absence of iodine-avid or mixed iodine-avid and non-avid lesions on 131-I Sodium iodide (NaI) whole-body scan, and 3) presence of FDG-avid lesions on the first 18F-FDG PET-CT scan. Patients with non-FDG-avid metastatic lesions that remained stable on anatomical imaging, stable Tg titer, and no identifiable changes in size or extension were considered to have stable disease.

Statistical analysis

Continuous variables were reported as means with standard deviation (SD), and dichotomous variables as percentages. The student t-test and the Fisher exact test were employed to compare imaging parameters (TLG, TLV) and biomarker variables (Tg and TgDT) between patients with and without disease progression. The hazard ratio (HR)of variables on PFS was assessed using a univariate Cox regression analysis between mean and threshold values of TLV, TLG, SUVmax, Tg, and TgDT and PFS. Subsequently, a multivariate Cox analysis was employed for the most statistically significant variables identified in the univariate Cox analysis to evaluate their impact on PFS. Finally, Kaplan-Meier log-rank analysis was used to evaluate the impact of various variables on PFS and visually represent this impact. SPSS software version 28.1.1 was used, with a significance level set at P < 0.05.

## Results

Baseline characteristics

A total of 23 patients were reviewed, with one patient excluded from the analysis as all their 18F-FDG PET-CT scans were normal. Among the remaining 22 patients, 15 (68%) were male. The mean patient age at the time of the 18F-FDG PET-CT scan was 63 years (range: 38 to 83 years).

Baseline imaging results

A total of 95 baseline and follow-up 18F-FDG PET-CT scans were evaluated from the 22 RR-DTC cases. At baseline, 13 out of 22 (59.1%) patients demonstrated PET-CT distant metastatic disease, while 9 out of 22 (40.9%) patients showed local cervical lymph node involvement. Among these patients, 11 out of 22 (50%) had subsequent disease progression, while the rest showed stable disease.

The FDG parameters were extracted from the scan at the time of diagnosis of RR-DTC, and follow-up 18F-FDG PET-CT scans were used to confirm disease progression or stability alongside other parameters.

The number of lesions per patient evaluated by metabolic parameters in this study ranged from 1 to 9. The follow-up time ranged from 21 to 452 months, with a mean follow-up of 116 months. The time to disease progression in the progressive disease cohort ranged from 4 to 452 months, with a median time to disease progression of 74 months. The 5-year PFS in our cohort was estimated to be 27%. None of the patients in the study died during the observation period.

The SUVmax of the lesions ranged from 2.0 to 64.3, with a mean ± SD of 14.5±16. The SUVmax among patients with progressive disease was higher than those with stable disease, with SUVmax ± SD values of 23.2±19.9 vs. 5.7±4. The difference was statistically significant with a p-value of <0.001 (t-value: -3.99). The SUVmax among patients with metastatic disease was higher, with a mean SUVmax ± SD of 19 ± 20 vs. 8±7.06 for local disease; the difference was statistically significant with a p-value <0.001(t-value: -3.94).

TLV among our cohort varied, ranging from 0.2 to 24.9 mL, with a mean ± SD of 4.6±6.8 mL. TLV was higher among progressive diseases compared to stable disease, with a mean TLV of 7.4 ± 8.9 mL compared to 1.7 ± 1.1 mL, respectively. The difference was significant with a p-value of 0.009 (t-value: -2.87). TLV was also higher among metastatic diseases, with a mean TLV of 6.7±8.3 mL compared to 1.5 ± 1.4 mL in local disease, which was statistically significant with a p-value of 0.01 (t-value: -2.79).

Regarding TLG, values ranged from 0.5 to 462.2, with a mean ± SD of 60.8±134. TLG was higher among progressive disease compared to stable disease, with a mean TLG of 115.6±176.2 for progressive disease compared to 5.9±4.7 for stable disease; the difference between the two categories meets statistical significance with a p-value of 0.45 (t-value: -2.06). The difference in TLG between metastatic disease (mean TLG of 98.8± 166.1) and local disease (mean TLG of 5.9±5.7) reached statistical significance, with a p-value of 0.047 (t-value: -2.11).

Biomarker results

Our data showed that the baseline mean and SD of Tg levels among our cohort were 70.4±199.4 ng/mL, ranging from 0.1 to 886 ng/mL. Tg levels were higher among the cohort with progressive disease compared to stable disease, with a mean Tg of 100.5 ± 262 ng/L compared to 40.4 ±113 ng/L, although the difference was not significant with a p-value of 0.114 (t-value: -1.65). Similarly, the mean Tg in metastatic disease was 113.7 ± 254.2 ng/L compared to 8.0 ± 6.0 ng/L in local disease; however, this difference did not meet the criteria for statistical significance, with a p-value of 0.11 (t-value: -1.64).

On the other hand, TgDT among our cohort ranged from 10 to 5710 days, with a mean ± SD of 453±1182.5 days. TgDT among groups with progressive diseases was shorter, with a mean TgDT of 138 days compared to 728 days among groups with stable diseases. However, this difference did not meet the criteria for significance, with a p-value of 0.22. TgDT among metastatic disease was 609 ± 1539.2 days, and for local disease, it was 228 ± 142.5 days; however, the difference was not statistically significant, with a p-value of 0.087 (t-value: -1.79).

Table 1 summarizes the pathological subtype, number of lesions, months of follow-up, disease status, imaging characteristics, and biomarker values for each patient in this study.

**Table 1:** Pathologic findings, anatomic location, treatment, metabolic, and biochemical characteristics of RR-DTC in this study Data are presented as individual patient values. Follow-up is reported in months. TLG (total lesion glycolysis) is calculated as SUV mean × volume (ml). Tg (thyroglobulin) is reported in nanograms per milliliter (ng/mL). TLV (total lesion volume) is reported in milliliters (mL). RAI (radioactive iodine ablation), and TgDT (thyroglobulin doubling time) are reported in days. Categorical variables are coded as follows: Metastatic: yes = 1, no = 0; Status: stable = 0, progressed = 1. RR-DTC: radioiodine-refractory differentiated thyroid cancer; pap ca: papillary thyroid cancer; dif.: differentiated

Follow-up Mo	Lesion no.	Metastatic yes=1	Location	Type of Cancer	Previous Treatment	Status Stable=0 Progressed=1	TLV	SUVmax	Tg	TLG	TgDT Days
48	1	1	Lung/mediastinum	Pap ca, follicular	Thyroidectomy, RAI, and lenvatinib	0	1.8	5.2	381.0	5.7	263
12	9	1	Mediastinum/neck	Pap ca, oncocytic	Thyroidectomy, RAI, and radiotherapy	1	18.2	64.3	0.9	462.2	51
86	6	1	Axilla/inguinal/neck	Pap ca, classic	Thyroidectomy and RAI	1	3.8	41.1	106.4	55.0	105
4	7	1	Mediastinum/neck	Pap ca, follicular	Thyroidectomy and RAI	1	24.9	32.4	29.1	225.9	10
396	1	1	Lung	Pap ca, classic	Thyroidectomy and RAI	0	1.8	2.0	3.6	2.3	558
53	1	0	Local	Pap ca, classic	Thyroidectomy and RAI	0	0.4	5.6	6.4	1.4	149
30	3	0	Local	Pap ca, tall cell	Thyroidectomy and RAI	0	2.0	6.0	2.2	7.3	71
279	3	1	Mediastinum	Pap ca, classic	Thyroidectomy and RAI	0	1.8	3.2	0.1	4.4	5,710
21	6	0	Local	Pap ca, tall cell	Thyroidectomy and RAI	0	3.5	2.7	11.8	6.5	462
22	3	1	Bone, neck	Pap ca, classic	Thyroidectomy and RAI	1	17.5	41.7	2.9	430.5	265
28	2	0	Local	Pap ca, oncocytic	Thyroidectomy and RAI	0	3.3	10.9	6.9	13.0	137
34	2	0	Local	Follicular ca, oncocytic	Thyroidectomy and RAI	1	2.6	12.2	21.4	16.8	110
28	1	0	Local	Pap ca, classic, and follicular	Thyroidectomy and RAI	0	0.3	3.4	9.4	0.9	359
216	1	0	Local	Pap ca, tall cell	Thyroidectomy and RAI	0	0.2	2.1	3.4	0.5	407
8	2	1	Mediastinum/neck	Pap ca, classic	thyroidectomy and RAI	0	2.2	6.0	4.4	8.3	138
75	1	1	Mediastinum/neck	Pap ca, classic	Thyroidectomy and RAI	1	0.3	4.2	12.2	1.2	253
36	7	1	Mediastinum/ neck	Pap ca, classic	Thyroidectomy, RAI, radiotherapy, and cabozantinib	1	10.5	21.6	13.9	67.0	215
23	5	1	Mediastinum/ neck	Pap ca, classic	Thyroidectomy, RAI, and radiotherapy	1	2.0	3.8	21.6	4.8	63
24	1	1	Bone, muscle	Pap ca, classic	Thyroidectomy and RAI	1	0.5	4.9	886.0	2.0	93
9	1	1	Bone	Pap ca, oncocytic, hurtle	Thyroidectomy and RAI	0	1.6	16.4	15.6	14.6	188
43	1	0	Local	Pap ca, follicular poorly dif, hurtle	Thyroidectomy and RAI	1	0.3	24.3	2.2	5.1	162
151	1	0	Local	Pap ca, classic	Thyroidectomy and RAI	1	0.8	4.4	8.7	1.8	197

Table 2 summarizes the baseline demographics and disease status of participants. 

**Table 2:** Baseline characteristics of the participants n: number of cases in that cohort, (%): percentage among that cohort, SD: standard deviation

Variables	All patients =22 n (%)	No progression=11 n (%)	Progression=11 n (%)
Number of participants	22(100%)	11(50%)	11(50%)
Male	15 (68%)	7 (64%)	8 (73%)
Female	7 (32%)	4 (36%)	3 (27%)
Variables	All patients mean ± SD	No progression mean ± SD	Progression mean ± SD
Participants age years	63 ± 13.1	64 ± 11.9	62 ± 14.7
Male participants’ age in years	64 ± 13.06	66.4 ± 12.2	63 ± 14.3
Female participants’ age in years	60 ± 13.7	60 ± 11.5	61 ± 19.1

Table 3 presents imaging and biochemical measures across cohorts, with statistical comparisons between disease subgroups.

**Table 3:** Comparison of various imaging and biochemical markers between groups n: number of cases in that cohort, (%): percentage among that cohort, SD: standard deviation, mL: milliliter, ng: nanograms, TLV: Total Lesion Volume, TLG: Total Lesion Glycolysis, TG: Thyroglobulin, TGDT: Thyroglobulin doubling time, SUV: Standardized Uptake Value; OR: Odds Ratio, ∞: infinity (due to presence of zero in categorical variable). A p-value of <0.05 was considered statistically significant.

Marker/ Variables	All Participants=22 n (%)	No Progression=11 n (%)	Progression=11 n (%)	P-value	Statistical Test	Value: Odds Ratio
Presence of metastasis	13 (59.1%)	5 (45.5%)	8 (72.7%)	0.387	Fisher’s Exact Test	3.20
TLV>5 mL	4 (18.2%)	0 (0%)	4 (36.4%)	0.0902	Fisher’s Exact Test	∞
TLG>2 (SUV mean*mL)	16 (72.7%)	8 (72.7%)	8 (72.7%)	1.00	Fisher’s Exact Test	1.0
TLG>20 (SUV mean*mL)	5 (22.7%)	0 (0%)	5 (45.4%)	0.0351	Fisher’s Exact Test	∞
TLG>100 (SUV mean*mL)	3 (13.6%)	0 (0%)	3 (27.3%)	0.0902	Fisher’s Exact Test	∞
TGDT≤100 days	5 (22.7%)	1 (9.1%)	4 (36.4%)	0.311	Fisher’s Exact Test	5.71
TGDT≥300 days	17 (77.3%)	6 (54.5%)	11 (100%)	0.0351	Fisher’s Exact Test	∞
TG>10 ng/m	9(40.9%)	2 (18.2%)	7 (63.6%)	0.198	Fisher’s Exact Test	4.67
TG>100 ng/mL	3 (13.6%)	1 (9.1%)	2 (18.2%)	1.00	Fisher’s Exact Test	2.22
SUV>5	13 (59.1%)	6 (54.5%)	7 (63.6%)	1.00	Fisher’s Exact Test	1.46
SUV>10	9 (41%)	2 (18%)	7 (63.6%)	0.08	Fisher’s Exact Test	7.88
Marker	All participants mean ± SD	No progression mean ± SD	Progression mean ± SD	P-value	Statistical Test	Value: t-value
Highest SUV	14.5±16.6	5.7 ± 4.3	23.2 ± 19.9	0.0006	Students’ T-test	-3.99
TLV (mL)	4.6±6.8	1.7 ± 1.1	7.4 ± 8.9	0.009	Students’ T-test	-2.87
TG (ng/mL)	70.4±199.4	40.4 ± 113	100.5 ± 262	0.114	Students’ T-test	-1.65
TLG (SUV mean*mL)	60.8±134	5.9 ± 4.7	115.6 ± 176.2	0.045	Students’ T-test	-2.06
TGDT (days)	453±1182.5	768 ± 1646	138 ± 85	0.087	Students’ T-test	-1.79

Predictive variables for disease progression

**Figure 1:**
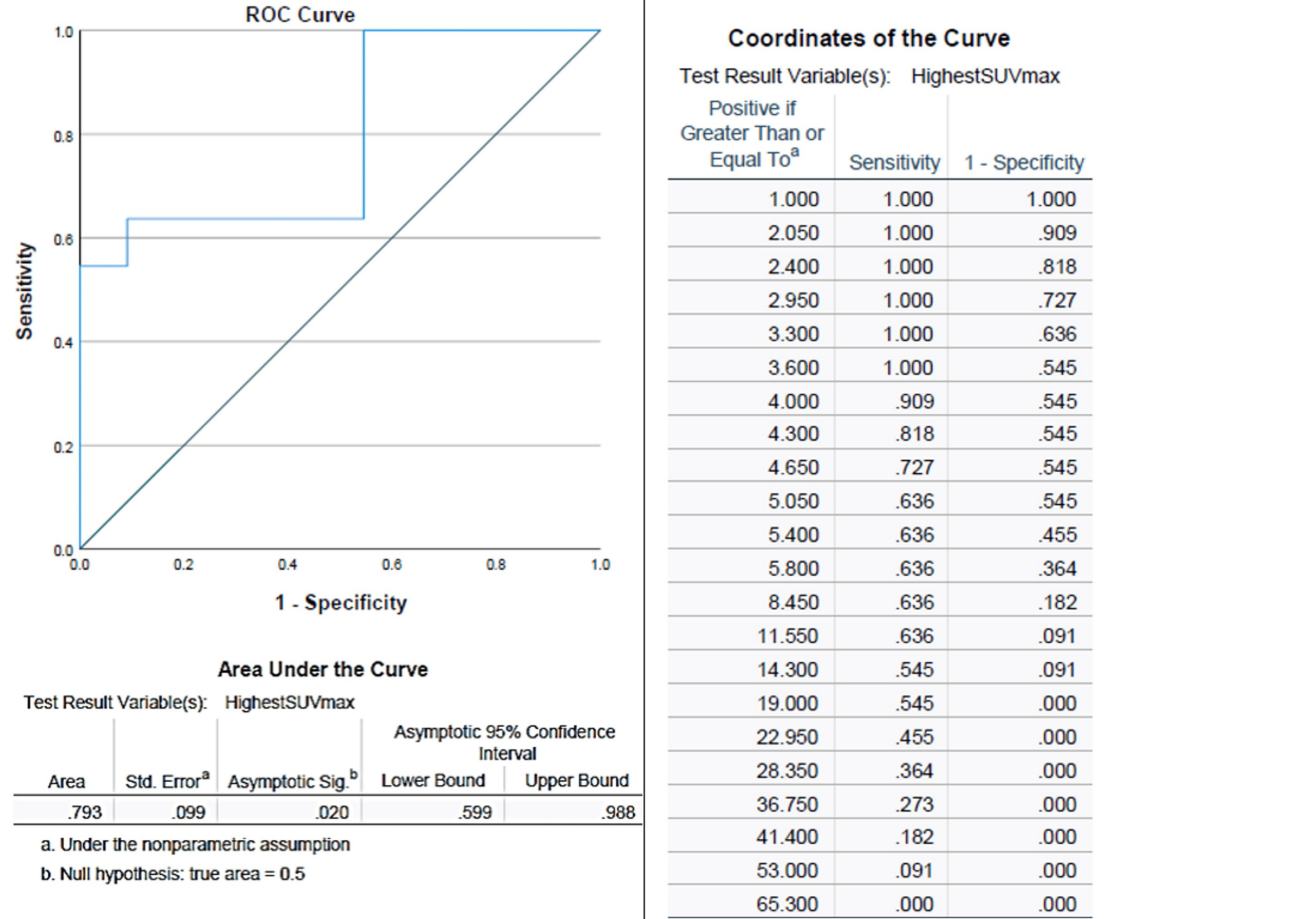
ROC curve of SUVmax among our cohort The receiver-operating characteristic (ROC) curve shows that SUVmax 12 can predict progression-free survival with a sensitivity of 0.64 and specificity of 0.91 (an area under the curve of 0.793).

The evaluation of SUVmax using univariate Cox regression demonstrated that an SUVmax of 10, with a hazard ratio (HR) of 4.97 (95% CI: 1.39-17.8), was statistically significant with a p-value of 0.014. Furthermore, a receiver operating characteristic (ROC) curve showed that an SUVmax cutoff of 12 yielded an area under the curve of 0.793, with a sensitivity of 0.64 and specificity of 0.91 for PFS (Figure 1).

Regarding the 18F-FDG PET-CT marker of TLV, the univariate Cox regression indicated that a TLV greater than 5 mL had an HR of 11.6 (95% C.I: 2.51-53.4) with a p-value of 0.002 in terms of PFS among RR-DTC patients.

A separate univariate Cox regression analysis demonstrated that even though the TLG value changes are meaningful between the two groups, the HR was not robust and the effect size was small, HR of 1.01 (95% C.I: 2.51-53.4) with a p-value of 0.009.

The mean of the biomarker Tg was not significantly different between progressive disease and stable disease in RR-DTC. However, univariate Cox regression analysis in our data revealed that a Tg value >10, with an HR of 5.70 (95% C.I: 2.51-53.4), was correlated with worse PFS and reached statistical significance with a p-value of 0.013.

A TgDT of 100 days or less, with an HR of 17.9, was found to be significant as a negative prognostic factor for PFS, with a p-value of 0.01.

In a multivariate Cox regression analysis among the variables with significant p-values and HR from the univariate Cox regression, only the TgDT of ≤ 100 demonstrated a significantly worse outcome, with an HR of 63.9 (95% CI: 9.33-743) and a p-value of 0.02. Additionally, TLV from the 18F-FDG PET-CT metabolic parameters also approached significance, with a p-value of 0.055 and an HR of 1.58 (95% CI: 0.99-2.53). Table 4 summarizes the results of the univariate Cox analyses.

PFS evaluation

Kaplan-Meier survival analyses were performed for various parameters used in univariate Cox regression analysis.

**Figure 2:**
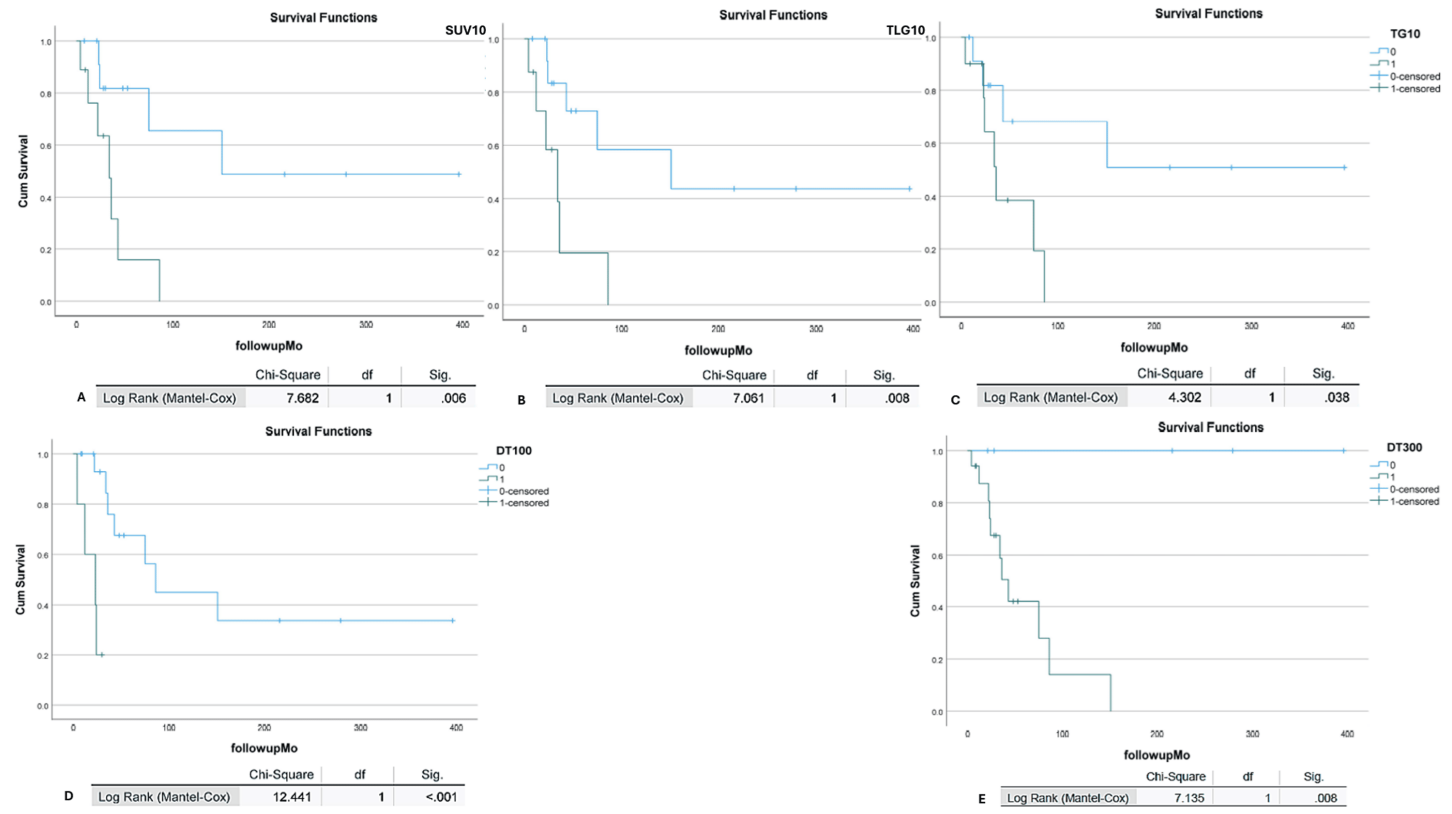
Kaplan-Meier survival analysis of PFS among our cohort A) SUVmax10 (0: ≤ 10 and 1:>10), B) TLG: total lesion glycolysis. TLG 10 (0: ≤ 10 and 1:>10), C) Tg: thyroglobulin. Tg 10 (0: ≤ 10 and 1:>10), D) DT: doubling time, thyroglobulin doubling time, TD100 (0: ≤ 100 days and 1:>100 days), and E) DT ≥300 thyroglobulin doubling time 300 (0: ≤ 300 days and 1:>300 days. Chi-Square value, degree of freedom (df) and p-values (sig.) are provided. A p-value <0.05 considered as significant). Kaplan-Meier analysis showed a worse PFS with SUVmax>10, TLG>10, TG >10, and TgDT ≤100 days, with the best PFS with TgDT ≥300 days.

The analysis for SUVmax ≤10 demonstrated a better PFS, with a p-value of 0.006 (Figure 2A). Among various TLG levels, the Kaplan-Meier survival analysis showed that TLG ≤ 10 had a better PFS of 151 months compared to 34 months for TLG greater than 10, with a p-value of 0.008 (Figure 2B). This was also evident in the Kaplan-Meier plot, where patients with Tg ≤ 10 had PFS of 237 months compared to 46.5 months for those with Tg greater than 10 ng/L, with a significant p-value of 0.038 (Figure 2C). A correlation between TgDT and PFS was assessed with Kaplan-Meier analysis, revealing that a TgDT of 100 days or shorter was related to worse PFS, with a p-value of <0.001 (Figure 2D). In contrast, a TgDT ≥ 300 days was correlated with better PFS (Figure 2E).

## Discussion

In thyroid cancer patients, the identification of RR-DTC is of paramount importance due to the limited treatment options available for managing this disease and its lower survival rates compared to DTC. RR-DTC occurs in approximately 5-15% of thyroid cancer patients during their illness [23] and is associated with a lower survival rate compared to DTC [24].

Suspected cases of radioiodine-refractory thyroid cancer can be confirmed through clinical and imaging modalities. Following the diagnosis of RR-DTC, the next crucial step is to identify patients who may exhibit earlier disease progression. This is imperative for devising improved treatment strategies. Functional imaging with 18F FDG PET-CT holds promise in the risk assessment of RR-DTC patients, allowing the identification of patients at a higher risk of rapid disease progression. Consequently, this subgroup of patients can benefit from the timely initiation of redifferentiation therapy, systemic therapy, or external beam radiation. Among the various measurements obtainable through 18F FDG PET-CT imaging in cancer patients, metabolic parameters such as SUV, TLV, and TLG have been evaluated in various diseases and cancers.

Several research studies have aimed to underscore the significance of metabolic parameters on prognosis and outcomes. For instance, a study by Kim et al. in those with thyroid cancer and lateral cervical lymph node metastatic disease, identified SUVmax of 5.91, MTV of 2.05 mL, and TLG of 9.09 as potential cut points for predicting lateral cervical neck metastasis. However, MTV and TLG did not prove to be valuable predictors of central neck lymph node involvement or extrathyroidal or multifocal disease [25].

In a study by Manohar et al. involving 62 patients diagnosed with metastatic RR-DTC, a 5-year overall survival (OS) probability of 34% and a 5-year PFS probability of 19% were observed. This study found that TSH-suppressed thyroglobulin (Tg) levels exceeding 100 ng/mL and a TgDT of less than 6 months were associated with worse outcomes in terms of OS and PFS. Additionally, higher-than-median values of MTV and TLG were correlated with worse OS and PFS. In their study, log-MTV and Log-TLG demonstrated higher hazard ratios for PFS and OS [21].

In contrast, our cohort showed no mortality during follow-up. The 5-year PFS among our cohort was estimated to be 27%, with a median time-to-progression of 74 months. These results were more favorable than those reported by Robbins et al., who demonstrated a PFS of 53 months [18]. However, the 10-year PFS in our data was lower, with only 4 out of 22 patients (18%) reaching this milestone. Nevertheless, it was higher than the figure reported by Durante et al., who found a 10-year OS of 10% in their cohort [23].

With respect to metabolic parameters, a study by Mason-Deshayes et al. involving 37 RR-DTC patients found that SUVmax < 10 and TLG < 154 had prognostic value with better PFS. MTV, on the other hand, did not exhibit a definite impact on predicting survival in DTC [26].

In a study by Gay et al., it was found that 18F FDG PET-CT metabolic parameters correlated with OS in patients who received TKIs. However, specific values or cutoff points were not provided [7].

Ye Liu et al. studied DTC and demonstrated that high uptake values in the primary lesion sites and metastases, with an SUVmax cutoff of 10, had an odds ratio (OR) of 39.5 for primary sites and an OR of 4.4 for metastatic sites. These findings suggest that these values can be integrated into a scoring system, along with age and early disease recurrence after surgery, to predict RR-DTC among DTC patients [27].

Roy et al. evaluated 34 RR-DTC patients from 2007 to 2019 and attempted to correlate MTV and SUVmax with disease progression in specific metastatic sites, including the neck, mediastinum, lungs, liver, bone, and total body metastatic sites. They observed SUVmax values of 5.3 and 3.9 in liver and mediastinal lesions, respectively, and a TLV of 1.16 cm³ in the liver and 79.5 cm³ for the total body metastatic sites volume, all of which correlated with worse outcomes [28].

Our study focused on 18F FDG PET-CT imaging metabolic parameters in correlation with Tg biomarker and TgDT to predict the behavior of RR-DTC. Based on our data, on univariate analysis, high uptake values with an SUVmax greater than 10, TLV greater than 5, and TLG greater than 10 with high HR can be considered parameters indicative of worse outcomes among our RR-DTC patients with shorter PFS. These variables, in conjunction with a Tg level > 10 ng/L and a TgDT of less than or equal to 100 days, correlated with worse outcomes and shorter PFS in our cohort. The Tg level among our cohort with progressive disease was higher compared to those with stable disease. Indeed, the mean Tg was higher in metastatic disease compared to local disease. These findings align with a previous study by Wassermann et al. on RR-DTC, which demonstrated higher Tg levels among iodine-negative DTC and progressive disease, with a mean Tg of 11.8 compared to 300 [29]. However, this difference did not reach statistical significance.

Conversely, the TgDT among our patients with progressive disease was shorter, with a mean TgDT of 138 days, compared to 728 days among those with stable disease. This finding is consistent with the results of Miyauchi et al. in a study evaluating TgDT in papillary thyroid cancer, where patients with a TgDT of less than 1 year demonstrated worse outcomes [30].

Strengths of this study include its integrative approach to prognostication in RR-DTC, combining metabolic parameters from 18F-FDG PET/CT (SUVmax, TLV, TLG) with serum thyroglobulin (Tg) levels and TgDT to stratify risk and predict PFS. This multimodal assessment allowed for the identification of specific thresholds, such as SUVmax >10, TLV >5, and TgDT ≤100 days, that correlated significantly with shorter PFS, thereby offering clinically actionable markers for early intervention. The results underscore the potential utility of including quantitative metabolic data in routine PET/CT reports to support individualized treatment decisions in this high-risk population.

Limitations and future work

This study has several limitations, including its retrospective design, single-center setting, and small sample size. To enhance the generalizability of these findings, a multicenter evaluation with a larger patient population would be beneficial. Additionally, our evaluation was performed at the onset of RR-DTC diagnosis and did not include an investigation into the impact of metabolic parameters on the response to systemic therapy, including TKIs. It would be intriguing to conduct follow-up studies on patients undergoing TKI treatment to explore the role of these same metabolic parameters in predicting treatment responses.

## Conclusions

RR-DTC represents a subgroup of thyroid cancer defined by limited treatment options, including TKIs, which may cause significant side effects. The metabolic characteristics observed in 18F-FDG PET-CT imaging can serve as valuable predictors of patient survival. In our cohort, we demonstrated differences in PFS when specific metabolic parameter thresholds were applied. Including these metabolic parameters in the report of an 18F-FDG PET-CT imaging study for patients with RR-DTC can assist referring physicians in the management of their patients.
